# Identification of FDA-Approved Drugs with Activity against Stationary Phase *Bartonella henselae*

**DOI:** 10.3390/antibiotics8020050

**Published:** 2019-04-29

**Authors:** Tingting Li, Jie Feng, Shuzhen Xiao, Wanliang Shi, David Sullivan, Ying Zhang

**Affiliations:** 1 Department of Molecular Microbiology and Immunology, Bloomberg School of Public Health, Johns Hopkins University, Baltimore, MD 21205, USA; litt@lzu.edu.cn (T.L.); jfeng@lzu.edu.cn (J.F.); zndxxsz@163.com (S.X.); wshi3@jhu.edu (W.S.); dsulliv7@jhmi.edu (D.S.); 2 Department of Immunology, School of Basic Medical Sciences, Lanzhou University, Lanzhou 730000, China; 3 Institute of Pathogenic Biology, School of Basic Medical Sciences, Lanzhou University, Lanzhou 730000, China; 4 Department of Clinical Microbiology, Ruijin Hospital, Shanghai Jiao Tong University School of Medicine, Shanghai 200025, China

**Keywords:** FDA-approved drug library, *Bartonella henselae*, persisters, stationary phase, antimicrobial activity

## Abstract

*Bartonella henselae* can cause various infections in humans, ranging from benign and self-limiting diseases to severe and life-threatening diseases as well as persistent infections that are difficult to treat. To develop more effective treatments for persistent Bartonella infections, in this study, we performed a high-throughput screen of an FDA-approved drug library against stationary phase *B. henselae* using the SYBR Green I/propidium iodide (PI) viability assay. We identified 110 drug candidates that had better activity against stationary phase *B. henselae* than ciprofloxacin, and among the top 52 drug candidates tested, 41 drugs were confirmed by microscopy to have higher activity than the current frontline antibiotic erythromycin. The identified top drug candidates include pyrvinium pamoate, daptomycin, methylene blue, azole drugs (clotrimazole, miconazole, sulconazole, econazole, oxiconazole, butoconazole, bifonazole), aminoglycosides (gentamicin and streptomycin, amikacin, kanamycin), amifostine (Ethyol), antiviral Lopinavir/ritonavir, colistin, nitroxoline, nitrofurantoin, verteporfin, pentamidine, berberine, aprepitant, olsalazine, clinafloxacin, and clofoctol. Pyrvinium pamoate, daptomycin, methylene blue, clotrimazole, and gentamicin and streptomycin at their respective maximum drug concentration in serum (C_max_) had the capacity to completely eradicate stationary phase *B. henselae* after 3-day drug exposure in subculture studies. While the currently used drugs for treating bartonellosis, including rifampin, erythromycin, azithromycin, doxycycline, and ciprofloxacin, had very low minimal inhibitory concentration (MIC) against growing *B. henselae*, they had relatively poor activity against stationary phase *B. henselae*, except aminoglycosides. The identified FDA-approved agents with activity against stationary phase *B. henselae* should facilitate development of more effective treatments for persistent Bartonella infections.

## 1. Introduction

Bartonella species are fastidious Gram-negative intracellular bacteria that are highly adapted to their mammalian reservoir hosts. Being the predominant cause of cat scratch disease in people and the second most common species causing endocarditis and bacillary angiomatosis, they have the ability to cause either acute or chronic infections and are responsible for different clinical conditions affecting humans [1]. The bacterium can be transmitted by cat’s scratch, and blood-sucking arthropod vectors such as fleas, sandflies, mosquitoes, or ticks and is considered to be an important opportunistic pathogen [2]. The bacterium can persist in the bloodstream of the host as the results of intraerythrocytic parasitism [3,4], and can cause a wide range of systemic disease such as bacteremia and central nervous system pathologies, especially in immunocompromised individuals [5]. *B. henselae* is sometimes a co-infection pathogen of Lyme disease transmitted by ticks carrying multiple pathogens causing more severe and protracted clinical manifestations [6]. It is very difficult to isolate and grow *B. henselae* in liquid media especially from clinical samples [7], as it is extremely fastidious, impeding progress in research of this organism. Thus, clinically, serology, and PCR are often used instead of culture to confirm diagnosis [7]. Treatment of systemic *B. henselae* infections has been difficult with poor clinical outcomes despite antibiotic treatment for weeks and months [8]. The first-line antibiotics for treatment of Bartonella infections include doxycycline, erythromycin, azithromycin, tetracyclines, gentamicin, rifampin, ciprofloxacin, and sulfa drugs, but most investigators have observed no benefit of antibiotic therapy for the treatment of typical, uncomplicated cat scratch disease [9,10], and effective treatment for Bartonella persistent infections remains a challenge. One possibility of the difficulty of eradicating *B. henselae* infections is due to bacterial persistence, a phenomenon that is well known to underlie persistent infections like tuberculosis, brucellosis, and Q-fever [11]. Previously, we successfully used the SYBR Green/PI viability assay for rapid high throughput drug screens for stationary phase *Borrelia burgdorferi* [12,13,14], which has been shown to be a good surrogate model of persister bacteria [15]. In this study, we adapted the same SYBR Green/PI methodology and performed a high throughput drug screen using stationary phase culture of *B. henselae* and identified a range of drug candidates from the FDA-drug library that have much better activity against the non-replicating *B. henselae* than the current antibiotics used to treat bartonellosis. The implication of finding such persister drugs for more effective treatment of persistent Bartonella infection is discussed.

## 2. Materials and Methods

### 2.1. Bacterial Strain, Culture Media, and Culture Conditions

The following reagent was obtained through BEI Resources (ATCC), NIAID, NIH: *Bartonella henselae* strain JK53, NR-31834. *B. henselae* was cultured in Schneider’s medium supplemented with 10% fetal bovine serum (FBS) as described [16,17]. Cultures were incubated without shaking at 37 °C, 5% CO_2_ at all times. The colony forming unit (CFU) counting was performed on Columbia sheep blood agar (BD Biosciences, California, USA) after serial dilutions.

### 2.2. Standard Curve of SYBR Green I/PI Assay for B. henselae

The SYBR Green I/PI assay for rapid viability assessment of *B. henselae* was carried out as we described previously, which we have successfully used for high throughput drug screens against stationary phase *B. burgdorferi* [12]. Briefly, we used 70% isopropyl alcohol killed *B. henselae* dead cells and live cells mixed in different proportions (10^7^ bacteria/mL) to validate the SYBR Green I/PI assay using a *B. henselae* 5-day stationary phase culture. The cells were added in 96-well plate, stained with SYBR Green I/PI mixture, and incubated at room temperature in the dark for 15 min. With excitation wavelength at 485 nm, the fluorescence intensities from green emission (535, 538, and 565 nm) and red emission (612, 635, and 650 nm) were measured using HTS 7000 plus Bioassay Reader (Perkin Elmer Inc., Waltham, MA, USA). The green/red fluorescence ratios were measured for each proportion of live/dead *B. henselae* as described [12].

### 2.3. Antibiotics and the FDA Drug Library

Antibiotics, including doxycycline, cefuroxime, miconazole, metronidazole, rifampin, and fluconazole, were purchased from Sigma Aldrich and were dissolved in appropriate solvents [18] to form stock solutions. All the antibiotic stocks were filter-sterilized by a 0.2 μm filter except the DMSO stocks. Then the stocks were diluted into 500 μM and stored at −20 °C. The JHCCL FDA-approved drug library was prepared in 10 mM stock solutions with DMSO. Drugs in the master plates were diluted with PBS to make 500 μM pre-diluted working plates. The pre-diluted drug plates were sealed and stored at −20 °C.

### 2.4. Microscopy Techniques

The SYBR Green I/PI dye was added to drug-treated or control *B. henselae* cell suspensions for assessing the viability by ratio of green/red fluorescence as described previously [12,13]. Samples on specimens or 96-well plates were examined by BZ-X710 All-in-One fluorescence microscope (KEYENCE, Inc., Osaka, Japan). The residual bacteria viability was confirmed by analyzing three representative images of the same bacterial cell suspension using fluorescence microscopy. BZ-X Analyzer and Image Pro-Plus software were used to quantitatively determine the fluorescence intensity.

### 2.5. Screening of FDA-Approved Drug Library against Stationary Phase B. henselae

For the high-throughput drug screen, 100 μL *B. henselae* cell suspension from a 7-day old stationary phase culture was added in 96-well plates. Each compound (10 μL, final concentration 50 μM) from the pre-diluted plate or pre-diluted stock was added to the cell suspension. Plates were sealed and placed in 37 °C incubator over of a period of 5 days. SYBR Green I/ PI viability assay was used to assess the live and dead cells after antibiotic exposure as described [13]. Briefly, 10 μL SYBR Green I (100× stock, Invitrogen, Waltham, MA, USA) and propidium iodide (PI, 600 μM, Sigma, St. Louis MO, USA) staining mixture was added to each well of the 96-well plate and mixed thoroughly. The plates were incubated at room temperature in the dark for 15 minutes followed by plate reading using microplate reader (HTS 7000 plus Bioassay Reader, Perkin Elmer Inc., Waltham MA, USA). The green/red (535 nm/635 nm) fluorescence ratio of each well was used for calculating the residual viability percentage with least-square fitting analysis as described previously [12]. All tests were run in triplicate.

### 2.6. Drug Exposure Assay 

A 5-day old *B. henselae* stationary phase culture was used for drug exposure experiments. The antibiotic exposure was carried out in 1.5 mL Eppendorf tubes over the course of 5 days at 37 °C without shaking. Then the cells were collected by centrifugation and rinsed twice with fresh Schneider’s medium followed by resuspension in 1 mL fresh Schneider’s medium. The cell suspension was serially diluted and plated on Columbia blood agar plates for viable bacterial counts (colony forming unit, CFU).

### 2.7. Minimum inhibitory concentration (MIC) Determination

The standard microdilution method was used to determine the minimum inhibitory concentration (MIC) needed to inhibit visible growth of *B. henselae* after a 5-day incubation period. *B. henselae* cells (1 × 10^6^) were inoculated into each well of 96-well microplate containing 90 μL fresh modified Schneider’s medium per well. Each diluted drug (10 μL) was added to the culture. All experiments were run in triplicate. The 96-well plates were sealed and incubated at 37 °C with 5% CO_2_ for 5 days. Cell proliferation was assessed using the SYBR Green I/PI assay and a Petroff-Hausser counting chamber after the incubation.

## 3. Results

### 3.1. Growth Behavior of B. henselae in Modified Schneider’s Medium

**Figure 1:**
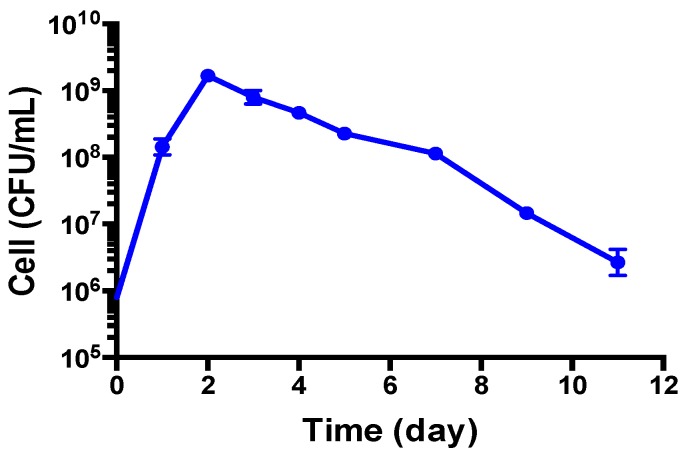
Growth of *B. henselae* (colony forming unit (CFU)/mL) in modified Schneider’s medium at 37 °C measured over a period of 11 days.

**Figure 2:**
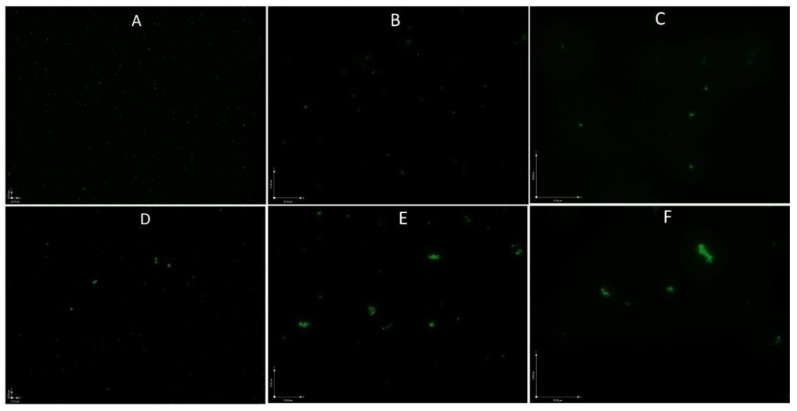
Fluorescence microscopic images of 1-day-old log phase cells (**A**–**C**) and 5-day-old stationary phase *B. henselae* cells (**D**–**F**). (**A**,**D**): 100 × magnification; (**B**,**E**): 400 × magnification; (**C**,**F**): 600 × magnification.

As the growth curve shows in Figure 1, *B. henselae* grew to logarithmic growth phase in 1 day and reached a growth peak at 2 days, with the highest CFU count reaching above 10^9^ per mL, followed by a decline to 10^8^ per mL at 5–7 days and a sharper decline to 10^6^ per mL at 11 days. However, when we checked the live and dead *B. henselae* cells after staining with SYBR Green I/ PI, all the visible cells in 1-day-old and 5-day-old *B. henselae* cultures fluoresced green under fluorescence microscope (Figure 2), indicating intact cell membranes. Interestingly, compared to the single, free-living cells in 1-day-old cultures (Figure 2A–C), many cells from 5-day-old cultures formed aggregated clusters (Figure 2D–F), which could partly contribute to the gradual drop of CFU count. Based on the growth curve (Figure 1) and the morphology of *B. henselae*, we considered 1-day-old culture as log phase culture and 5-day-old culture as stationary phase culture.

### 3.2. Development of a SYBR Green/PI Viability Assay for B. henselae

**Figure 3:**
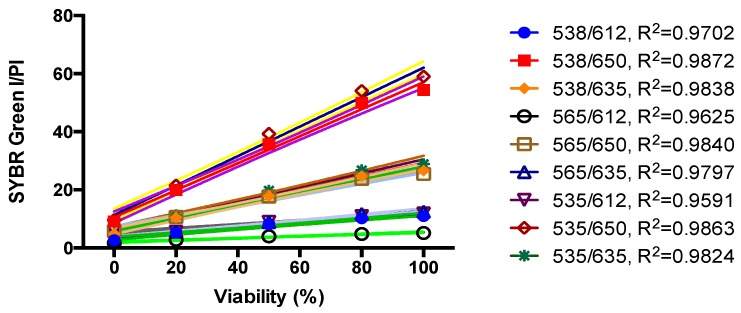
Linear relationship between the *B. henselae* viability and Green/Red fluorescence ratios of the SYBR Green I/PI assay. Various proportions of live and isopropyl alcohol-killed *B. henselae* were used to validate the SYBR Green I/PI assay on the 5-day old *B. henselae* stationary phase culture. Cell density was about 10^7^ bacteria/mL. The line is a least-squares fit of the relationship between the percentages of live bacteria and the green/red fluorescence ratios.

We previously developed a SYBR Green I/PI assay for rapid viability assessment of *Borrelia burgdorferi* [12] and have successfully used this assay for high throughput drug screens against non-growing stationary phase *B. burgdorferi* [13,14]. To perform high throughput drug screens on stationary phase *B. henselae*, here we adapted the SYBR Green I/PI methodology to *B. henselae*. As in our previous study [12], we used 70% isopropyl alcohol killed *B. henselae* dead cells and live cells mixed in different proportions (10^7^ bacteria/mL) to validate the SYBR Green I/PI assay using a *B. henselae* stationary phase culture. The results showed that the percentage of residual viability of different live and dead cell mixtures correlated well in linear relationships with the ratios of green/red fluorescence intensities of *B. henselae*, especially the ratio pairs of fluorescence intensities at 535 nm/650 nm and 538 nm/650 nm (Figure 3). This result indicates that the SYBR Green/PI assay could also be used for high throughput drug screens on *B. henselae*, and fluorescence intensities at 535 nm and 650 nm were used to calculate the ratio of green/red fluorescence in the SYBR Green I/PI assay of *B. henselae*.

### 3.3. Screening FDA Drug Library to Identify Drugs Active against Non-Growing Stationary Phase B. henselae Using the SYBR Green/PI Assay

As shown in Figure 1, the 5-day old *B. henselae* culture in modified Schneider’s medium could be considered to be in stationary phase and was thus used to identify active drugs against stationary phase *B. henselae*. We used the FDA drug library at 50 μM in the screen to increase the chance of obtaining hits as described previously [13]. Meanwhile, the currently known effective antibiotics used to treat bartonellosis, such as doxycycline, azithromycin, rifampin, ciprofloxacin, etc.; were included as control drugs for comparison (Table 1). In the primary screen, 110 of the 1581 drugs in the FDA drug library were found to have higher activity against stationary phase *B. henselae* than the control drug ciprofloxacin. According to our previous experience, some compounds can interfere with the SYBR Green I/PI assay because of color or autofluorescence. Thus, we selected 52 top active candidates for further validation by microscopic counting to confirm the SYBR Green I/PI plate reader results. Doxycycline and erythromycin, the most frequently used antibiotics for the treatment of Bartonella infection in humans, showed poor activity against stationary phase *B. henselae* (residual viability above 60%) (Table 1). Gentamicin, streptomycin, azithromycin, tetracycline, and rifampin, which are clinically used for treating Bartonella infections [19], had relatively better activity (residual viability between 32% and 59%) against the stationary phase *B. henselae* than doxycycline and erythromycin. Other control drugs proposed for Bartonella treatment, including penicillin, chloramphenicol, cefuroxime, and ciprofloxacin, did not show higher activity compared with the most frequently used doxycycline and erythromycin, with residual viability ranging from 60% to 74%.

From the 52 selected candidates that showed good activity against stationary phase *B. henselae* in the primary screen, we confirmed that 41 drugs showed higher activity than erythromycin (Table 1). Among the 41 active drugs, 7 azole drugs including sulconazole, econazole, oxiconazole, butoconazole, clotrimazole, bifonazole, and miconazole, showed high activity against stationary phase *B. henselae*. We found daptomycin, a powerful anti-persister drug against B. burgdorferi that we identified in our previous study [13], also showed strong activity (residual viability 21%) against stationary phase *B. henselae* (Table 1). In addition, methylene blue, another active drug against *B. burgdorferi* we identified before [14], was found to have good activity (residual viability 25%) against stationary phase *B. henselae* (Table 1). Furthermore, amifostine (Ethyol), Lopinavir/ritonavir, colistin, nitroxoline, berberine, amikacin, kanamycin, verteporfin, pentamidine, aprepitant, clinafloxacin, and clofoctol also showed relatively high activity against stationary phase *B. henselae* (Table 1). Pyrvinium pamoate exhibited excellent plate reader results in the primary screening, yet microscopic checking indicated this was due to strong autofluorescence background. The MIC measurement and subculture test of pyrvinium pamoate together with other representative drugs were carried out below to further confirm their activity against both growing and non-growing stationary phase *B. henselae*.

### 3.4. MIC Determination of Active Hits

While we found agents that had good activity against the non-growing stationary phase *B. henselae* (Table 1), it is also necessary to determine the MICs of the active hits against growing *B. henselae*. The MICs for *B. henselae* were determined by the standard microdilution method as described in our previous study [13]. As shown in Table 2, rifampin was the most active agent, capable of inhibiting *B. henselae* proliferation at the lowest concentration of rifampin tested (0.01 μg/mL).

The growth of *B. henselae* was efficiently suppressed by azithromycin and pyrvinium pamoate at a concentration of 0.04–0.08 μg/mL, by methylene blue, doxycycline and erythromycin at 0.08–0.16 μg/mL, and by clinafloxacin at 0.16–0.31 μg/mL. Nitrofurantoin, nitroxoline, and pentamidine were active with MIC values of 0.31–0.63 μg/mL. *B. henselae* growing cells were also susceptible to clotrimazole, gentamicin, and berberine with MICs of 0.63–1.25 μg/mL, ciprofloxacin with MIC of 1.25–2.5 μg/mL, streptomycin and miconazole with MIC of 3.13–6.25 μg/mL, and amikacin with MIC of 6.25–12.5 μg/mL. Aprepitant and clofoctol could inhibit growth of *B. henselae* with MIC of 10–20 μg/mL and 70–140 μg/mL, respectively, which are much higher than those corresponding maximum drug concentration in serum (C_max_) in human. Interestingly, daptomycin, which is highly active against non-growing stationary phase *B. henselae* (Table 1), had relatively poor activity against growing *B. henselae* with a high MIC of 12.5–25 μg/mL. Colistin and amifostine were the least effective agents in inhibiting the growth of *B. henselae*, with MICs higher than 80 μg/mL, which is more than 10 times higher than C_max_ for each agent.

### 3.5. Subculture Study of Stationary Phase B. henselae After Drug Exposure

Having obtained many candidates from the FDA drug library, we performed a time-kill drug exposure assay against a 5-day-old *B. henselae* stationary phase culture at concentrations of their respective C_max_. As shown in Table 3, pyrvinium pamoate, methylene blue and daptomycin were the most active agents, which rapidly killed *B. henselae* with no detectable CFU after 1-day exposure. Other active hits, including clotrimazole, gentamicin, and streptomycin, could lead to eradication of *B. henselae* cells without viable cells being recovered after exposure for 3 days. Nitroxoline also showed excellent activity, reducing 4 log10 CFU/mL after 3-day exposure. Clinafloxacin and nitrofurantoin also had the capability of killing stationary phase *B. henselae* and reduced the bacterial count by approximately 2 log10 CFU/mL in 3 days. However, although rifampin was the most effective agent against the growing *B. henselae* (Table 2), it failed to eradicate stationary phase *B. henselae* cells after treatment for 3 days, leaving about 8 × 10^3^ per mL CFU remaining (Table 3). Compared with control without drug exposure, clinically used antibiotics for treatment of Bartonella infections, including azithromycin, doxycycline, and erythromycin, had poor activity in killing *B. henselae*, achieving approximately 1 log10 decrease of CFU/mL after 1-day drug exposure and no obvious further decrease even after 3-day exposure. Other drugs, including pentamidine, berberine, ciprofloxacin, aprepitant, clofoctol, colistin, and amifostine, at their C_max_ values had poor activity against stationary phase *B. henselae*, with viable cells barely reduced.

### 3.6. Comparison of Susceptibility of Log Phase B. henselae and Stationary Phase B. henselae in Drug Exposure Assay

**Figure 4:**
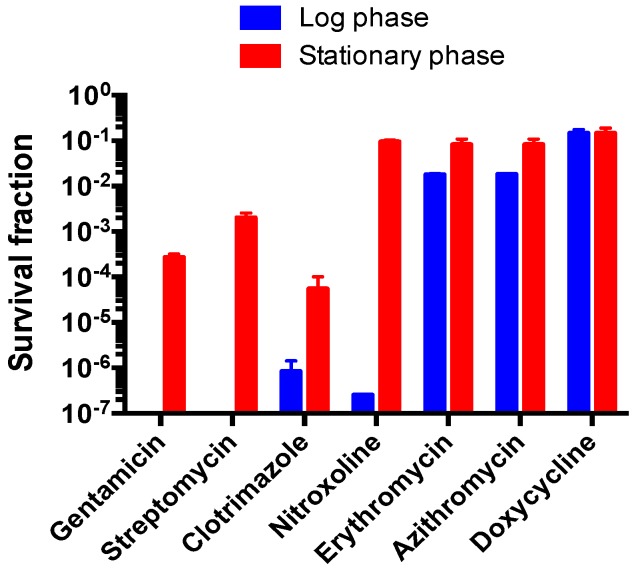
Comparison of survival fraction of a 1-day-old log phase *B. henselae* culture and a 5-day-old stationary phase *B. henselae* culture after drug exposure for 1 day. Survival fractions were calculated as: CFU/mL after drug exposure divided by CFU/mL in the control without drug. Drug concentrations used in this experiment are listed as follows: 10 μg/mL gentamicin, 25 μg/mL streptomycin, 25 μg/mL clotrimazole, 5 μg/mL nitroxoline, 1 μg/mL erythromycin, 2 μg/mL azithromycin and 5 μg/mL doxycycline.

To further demonstrate the divergent drug tolerance of *B. henselae* at different growth stages, we tested the efficacy of the top 7 drug hits screened from subculture test of 5-day-old stationary phase *B. henselae* after drug exposure, including pyrvinium pamoate, methylene blue, daptomycin, clotrimazole, gentamicin, streptomycin, and nitroxoline, against 1-day-old log phase and 5-day-old stationary phase *B. henselae* and compared the survival fraction of *B. henselae* cells within these two different growth stages. Similar to the results of subculture test for stationary phase *B. henselae* drug exposure experiment, pyrvinium pamoate, methylene blue, and daptomycin were the most active agents against log phase *B. henselae*, with no colony being detected on agar plate after treatment for 1 day and 3 days (data not shown). While gentamicin and streptomycin could lead to no viable cells in the log phase *B. henselae* culture after drug treatment for 1 day and 3 days (Figure 4), the survival fractions of the 5-day-old stationary phase *B. henselae* were around 10^−3^ and 10^−2^ after 1-day treatment with gentamicin and streptomycin, respectively. Clotrimazole could effectively kill stationary phase *B. henselae* cells, with 10^−4^ of 5-day-old stationary phase *B. henselae* cells and 10^−6^ of 1-day-old log phase *B. henselae* cells surviving after 1-day treatment. Although nitroxoline showed limited efficacy in killing stationary phase *B. henselae*, with around 10% of cells surviving after treatment, it could efficiently kill log phase *B. henselae*, with surviving fraction lower than 10^−6^. Furthermore, erythromycin and azithromycin used for treating Bartonella related diseases, did not show strong activity against stationary phase *B. henselae* cells with 10% of surviving fraction, yet both drugs could eliminate 99% of log phase *B. henselae* cells. Doxycycline had poor activity against both log phase and stationary phase *B. henselae* cells (Figure 4).

**Figure 5:**
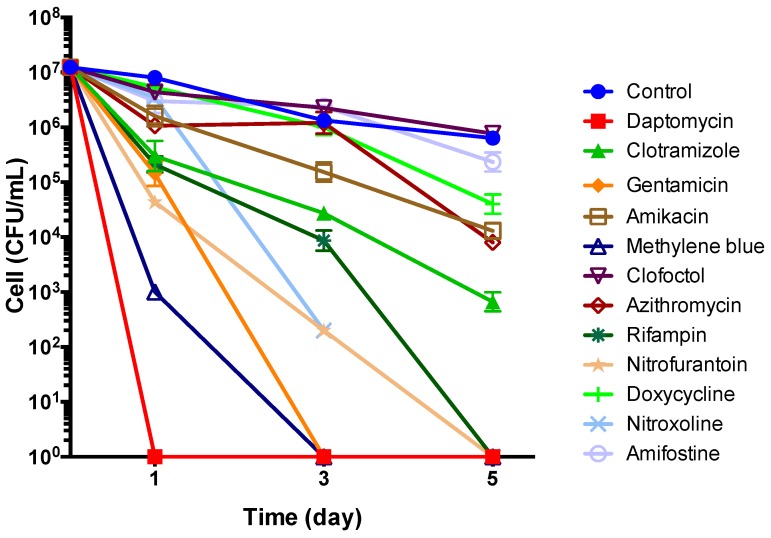
Time-kill curves for drug treatment of 5-day old stationary phase *B. henselae*. The antibiotics were added to the culture at time point 0, and at different times of drug exposure (Day 1, Day 3, and Day 5), portions of bacteria were removed and washed and plated on Columbia blood agar for CFU counts. Drug concentrations used in this experiment were based on their C_max_ (Table 2) and are as follows: 60 μg/mL daptomycin, 5 μg/mL clotrimazole, 5 μg/mL gentamicin, 5 μg/mL amikacin, 10 μg/mL methylene blue, 2 μg/mL clofoctol, 2 μg/mL azithromycin, 10 μg/mL rifampin, 5 μg/mL nitrofurantoin, 5 μg/mL doxycycline, 5 μg/mL nitroxoline, and 5 μg/mL amifostine.

In the time-kill experiment with 5-day old stationary phase *B. henselae* culture, the top 8 drug candidates (daptomycin, methylene blue, clotrimazole, gentamicin, nitrofurantoin, nitroxoline, clofoctol, amifostine) along with control drugs (rifampin, doxycycline, azithromycin, amikacin) at their C_max_ were added to the culture for different times of drug exposure (Day 1, Day 3, and Day 5) followed by CFU count. Daptomycin was found to be most active at killing all bacteria by Day 1, followed by methylene blue and gentamicin which killed all bacteria by Day 3 (Figure 5). Nitrofurantoin and rifampin were also quite active and killed all bacteria by Day 5, while clotrimazole at (5 ug/mL, close to Cmax) still had about 10^3^ bacteria remaining by Day 5. Amikacin, azithromycin, and doxycycline had some activity, whereas clofoctol and amifostine had poor or no activity (Figure 5).

## 4. Discussion

In this study, we were able to culture *B. henselae* in a reliable and consistent manner using a modified Schneider’s medium, which allowed 1-day-old culture to grow as log phase and 5-day-old culture as stationary phase (Figure 1). We then successfully adapted the SYBR Green/PI viability assay to *B. henselae* and performed a high throughput screen with FDA-approved drug library for activity against stationary phase *B. henselae*. We found that a significant number of drugs have better activity than the current drugs used to treat bartonellosis. These include seven azole drugs (sulconazole, econazole, oxiconazole, butoconazole, clotrimazole, bifonazole, and miconazole), daptomycin, methylene blue, amifostine (Ethyol), Lopinavir/ritonavir, colistin, amikacin, nitroxoline, berberine, verteporfin, pentamidine, aprepitant, clinafloxacin, and clofoctol. We then chose some of these drugs from different categories at appropriate concentrations to determine their MICs and capability of killing 5-day-old stationary phase *B. henselae*. The results demonstrated that *B. henselae* was highly susceptible to most antibiotics tested, which is consistent with previous reports [19,42]. However, the MICs did not correlate well with results of drug exposure against 5-day-old stationary phase *B. henselae*. Rifampin, which is the most effective agent in inhibiting growing *B. henselae* with the lowest MIC (Table 2), could not kill all stationary phase *B. henselae* cells. Furthermore, clinically used antibiotics for Bartonella infections, including azithromycin, doxycycline, and erythromycin, while exhibiting effective inhibition of growing *B. henselae* cells (Table 2), had very limited activity against aggregated *B. henselae* cells in stationary phase (Table 3).

Fortunately, 6 agents, pyrvinium pamoate, methylene blue, daptomycin, clotrimazole, gentamicin, and streptomycin, could efficiently kill stationary phase *B. henselae*, without CFU detected after drug exposure with drug concentrations close to C_max_ (Table 3). Among them, aminoglycosides gentamicin and streptomycin have been reported to have clinical improvement for treating Bartonella infection. Bartonella species have the capability to reside and propagate in erythrocytes in humans and animals [43], which probably provide a shelter for Bartonella sp.; protecting them from the host immune responses and exposure to antibiotics. Gentamicin is documented to enter erythrocytes slowly and poorly, with a peak level of 0.26 μg/mL [44], which is much lower than its MIC of 0.63–1.25 μg/mL. Thus, Bartonellae residing within erythrocytes are protected from gentamicin, and its clinical effect may be due to its activity against extracellular organisms.

Pyrvinium pamoate, an antihelmintic drug, had good activity against stationary phase *B. henselae*. While it is safe to take orally even with a high dose, pyrvinium pamoate could be barely detected in plasma and urine due to poor bioavailability [22]. One limitation to pyrvinium pamoate’s application is its poor absorption for humans. Attempts of structural modification should be taken to improve pyrvinium pamoate’s bioavailability, which could make it a potentially useful agent for targeting Bartonella infections due to its high activity against both growing and non-growing form of the organism.

Surprisingly, methylene blue showed great activity against *B. henselae*. It is interesting to note that we have previously found methylene blue has good activity against stationary phase Borrelia burgdorferi [14]. Methylene blue is a dye and a medication, which was used as an antimalarial agent and has received renewed attention in the management of other diseases, such as urinary tract infections (UTIs) and methemoglobinemia [45]. Methylene blue was recently shown to have antifungal effect through its effect on redox and membrane disruption [46]. It is interesting to note that membrane is a target of persister drugs, and our previous finding that methylene blue also had activity against Borrelia burgdorferi stationary phase cells [14] is consistent with its activity on the membrane. Further studies are needed to determine if methylene blue could disrupt membranes of *B. henselae*.

It is worth noting that we found daptomycin had excellent activity against stationary phase *B. henselae* (Table 3), although it had a high MIC (12.5–25 µg/mL) against growing organisms (Table 2). Interestingly, the powerful killing action of daptomycin was also observed in drug treatment of spirochete B. burgdorferi in our previous studies [13,47]. Daptomycin is a cyclic lipopeptide antibiotics with a broad spectrum of activity against Gram-positive bacteria and has been used to treat severe infections caused by antibiotic-resistant strains. It has calcium-dependent bactericidal activity by creating pores on bacterial cell membranes, leading to release of potassium, membrane potential dissipation, and ultimately cell death [48]. It is well known that daptomycin has no activity against Gram-negative bacteria due to the less anionic phospholipids in the Gram-negative cytoplasmic membrane compared to Gram-positive bacteria, which would result in insufficient sites for calcium-mediated insertion of daptomycin [49]. As far as we know, this is the first time that daptomycin has been shown to exhibit excellent activity against stationary phase *B. henselae*, a Gram-negative bacterium. The high activity of daptomycin against both Borrelia and Bartonella indicates it may serve as a promising drug candidate in treatment of both persistent Borrelia infections and Bartonella infections, which clinically may be present as coinfections.

It is interesting to note we found several antifungal azoles to have good activity against *B. henselae*. Clotrimazole at a therapeutically relevant concentration had excellent activity against both growing and non-growing *B. henselae* and could kill stationary phase *B. henselae* without viable cells detected on agar plates. In addition, it is of interest that clotrimazole has been shown to be clinically useful for treating other diseases, such as sickle cell anemia, malaria, beriberi, tinea pedis, Chagas disease, and cancer [50]. Clotrimazole acts as an anti-malarial agent by causing heme-induced membrane damage in the malaria parasite [51]. As a well-known antifungal agent, clotrimazole works by altering the permeability and fluidity of the fungal cell membrane through inhibition of ergosterol synthesis, with ergosterol depleted and replaced with unusual sterols [52]. There is no ergosterol synthesis in *B. henselae*, and the mechanisms of action of azoles, including clotrimazole, against *B. henselae* remains to be determined.

It is worth noting that discrepant efficacies of antibiotics exist between in vitro testing based on MIC data and clinical data in patients with Bartonella-related infections [8]. One contributing factor could be the existence of bacterial aggregates or biofilm in patients [43,53] or the aggregated clusters or biofilm structures or persisters we found in stationary phase cultures. While clinically used antibiotics for Bartonella infections, including rifampin, azithromycin, doxycycline, and erythromycin, could effectively inhibit the growth of the *B. henselae* strain (Table 2), they showed poor capability in eradicating stationary phase *B. henselae* cells in aggregated form (Table 3). In addition, gentamicin, streptomycin, clotrimazole, and nitroxoline, capable of wiping out or efficiently eliminating log phase *B. henselae* cells, had less activity against stationary phase *B. henselae* cells (Figure 4). Erythromycin and azithromycin frequently used in treatment of Bartonella-related infections, had limited activity against stationary phase *B. henselae* cells, but it had high activity against log phase *B. henselae* cells (Figure 4). The poor activity of the current clinically used drugs for treating bartonellosis against non-growing stationary phase *B. henselae* could partly be responsible for the treatment failure and relapse of bartonellosis [8]. Future studies are needed to determine if the drugs we identified in this study with activity against non-growing stationary phase *B. henselae* cells could be more effective at treating persistent infections in vivo in animal models and in patients in the context of drug combinations.

Another important factor that could lead to the discrepancies could be the localization of Bartonella in the host during the infection. The bacteria are able to reside and propagate inside erythrocytes and/or endothelial cells [43,53]. The intracellular localization of Bartonella provides a shelter for them to evade exposure to antibiotics, such as gentamicin, which is mainly active against extracellular bacteria but not intracellular bacteria. Thus, antibiotics with good activity against Bartonella in vitro should also be tested for their activity against intracellular bacteria in future.

While we found many promising drug candidates with good activity against stationary phase *B. henselae*, they may not be used alone due to possible resistance development and need to be used in the context of drug combinations. Future studies are needed to evaluate drug combinations using the newly identified drug candidates with the current drugs used in clinic to better target diverse bacterial populations in different niches that can happen in the host as in the Yin-Yang model [11]. In addition, our findings in vitro will require further validation using appropriate animal models of bartonellosis to assess the utility of the identified drug candidates for more effective treatment of persistent Bartonella infections in vivo and in the clinic. While our study was performed with *B. henselae*, the findings may apply to other closely related pathogenic Bartonella species, such as *B. quintana* and *B. bacilliformis*. Future studies are needed to confirm this possibility.

## 5. Conclusions

In summary, this is the first study of a high throughput drug screen against stationary phase *B. henselae* using the FDA-approved drug library where we identified a range of promising drug candidates that may improve the treatment of Bartonella infections. Since these drug candidates are FDA-approved they could be more readily adopted clinically for treating Bartonella infections as long as they meet appropriate safety, PK, and efficacy requirement. Further studies are needed to determine the activity of persister drug combinations with the identified drug candidates against Bartonella persisters and biofilm bacteria in vitro and in animal models of Bartonella infection.

## Figures and Tables

**Table 1:** Activity of top 41 active hits that had good activity against stationary phase *B. henselae* ^a^.

Drugs (50 μM)	Residual Viable Cell Percentage	
	Primary Screen ^b^	Confirmation Microscopy ^c^
Drug free control	60%	81%
**Gentamicin**	2%	32%
**Azithromycin**	17%	42%
**Streptomycin**	9%	39%
**Tetracycline**	12%	50%
**Rifampin**	2%	59%
**Doxycycline**	11%	60%
**Penicillin**	13%	60%
**Erythromycin**	14%	66%
**Chloramphenicol**	13%	70%
**Cefuroxime**	21%	73%
**Ciprofloxacin**	30%	74%
Pyrvinium pamoate	0% ^d^	ND ^e^
Amifostine	27%	10%
Daptomycin	7%	21%
Lopinavir/ritonavir	9%	21%
Sulconazole	3%	22%
Econazole	8%	22%
Oxiconazole	3%	24%
Butoconazole	11%	25%
Methylene blue	16%	25%
Clotrimazole	7%	27%
Dibekacin *	9%	28%
Abamectin *	2%	30%
Colistin	4%	30%
Bifonazole	4%	31%
Amikacin	12%	32%
Nitroxoline	6%	33%
Miconazole	8%	35%
Chlorosalicylanilide *	17%	38%
Berberine	8%	39%
Meclocycline *	11%	40%
Nebramycin *	2%	41%
Kanamycin	9%	44%
Dichlorophen *	14%	45%
Verteporfin	0% ^d^	45%
Pentamidine	15%	46%
Cloxyquin *	7%	48%
Aprepitant	0% ^d^	49%
Puromycin *	6%	49%
Amaranth *	3%	50%
Lomerizine ^*^	8%	51%
Carbomycin *	16%	52%
Spiramycin *	14%	53%
Thiethylperazine	4%	55%
Clinafloxacin	11%	56%
Clofoctol	12%	58%
Meclizine	11%	60%
Pazufloxacin *	12%	62%
Nitrofurantoin	16%	63%
Diclazuril *	15%	64%
Olsalazine	18%	64%
Nifuroxazide *	7%	65%

* Drugs are currently not listed in FDA-approved agents for human use. ^a^ Stationary phase *B. henselae* (5-day old) cells were treated with drugs for 48 hours. Currently used antibiotics for bartonellosis as controls are shown in bold. ^b^ Residual viability was calculated according to the regression equation and the ratio of Green/Red fluorescence obtained by SYBR Green I/PI assay. ^c^ Residual viability was assayed by epifluorescence microscope counting. ^d^ Values of SYBR Green I/PI are lower than the 100% dead cells. ^e^ Too strong autofluorescence background.

**Table 2:** Minimal inhibitory concentrations (MICs) of select drug candidates against *B. henselae* ^a^.

Antibiotics	MIC (μg/mL)	C_max_ (μg/mL)
Rifampin	0.01	15.6 [20]
Azithromycin	0.04–0.08	0.57 ± 0.23 [21]
Pyrvinium pamoate	0.04–0.08	0.003 * [22]
Methylene blue	0.08–0.16	3.91 ± 1.60 [23]
Doxycycline	0.08–0.16	1.5–7.0 [24]
Erythromycin	0.08–0.16	1.44 [25]
Clinafloxacin	0.16–0.31	5.0 [26]
Nitrofurantoin	0.31–0.63	0.88–0.96 [27]
Nitroxoline	0.31–0.63	5.59 ± 3.15 [28]
Pentamidine	0.31–0.63	0.22 ± 0.05 [29]
Clotrimazole	0.63–1.25	0.5–1.5 [30]
Gentamicin	0.63–1.25	11.0 ± 0.6 [31]
Berberine	0.63–1.25	0.00044 ± 0.00041 [32]
Ciprofloxacin	1.25–2.5	1.97–5.39 [33]
Streptomycin	3.13–6.25	29.52 [34]
Miconazole	3.13–6.25	6.26 [35]
Amikacin	6.25–12.5	101 ± 49.4 [36]
Aprepitant	10–20	3.07 ± 0.85 [37]
Daptomycin	12.5–25	55–133 [38]
Clofoctol	70–140	38.1 [39]
Colistin	80	1.21–3.36 [40]
Amifostine	160	16.99–19.89 [41]

^a^ The MIC testing for *B. henselae* was set up as described in Methods. C_max_: Maximum drug concentrations in serum were from the literature. NA: Not available. * Plasma level of pyrvinium (pyrvinium chloride equivalents) following administration of a single oral 250 mg dose of pyrvinium pamoate suspension (minimal detectable concentration of 0.0023 μg/mL).

**Table 3:** Evaluation of select drug candidates against a 5-day old stationary phase *B. henselae* culture at their respective maximum drug concentration in serum (C_max_) values.

Antimicrobial Agents	Con. of Drug Exposure (μg/mL)	CFU per mL after Drug Exposure	
		1 Day	3 Day
Control *	0	3.67 ± 2.08 × 10^7^	1.33 ± 0.11 × 10^6^
Rifampin	10	2.10 ± 0.85 × 10^5^	8.67 ± 0.46 × 10^3^
Azithromycin	2	3.00 ± 1.00 × 10^6^	5.33 ± 2.31 × 10^5^
Doxycycline	5	5.33 ± 1.53 × 10^6^	1.00 ± 0.40 × 10^6^
Erythromycin	1	3.00 ± 1.00 × 10^6^	1.00 ± 0.20 × 10^6^
Ciprofloxacin	5	1.77 ± 0.45 × 10^6^	2.60 ± 1.40 × 10^5^
Gentamicin	10	1.00 ± 0.17 × 10^4^	0
Streptomycin	25	7.33 ± 2.08 × 10^4^	0
Amikacin	100	2.00 ± 1.73 × 10^3^	0
Methylene blue	5	0	0
Daptomycin	60	0	0
Pyrvinium pamoate	5	0	0
Clotrimazole	25	2.00 ± 1.73 × 10^3^	0
Nitroxoline	5	3.47 ± 0.31 × 10^6^	2.00 ± 0.00 × 10^2^
Nitrofurantoin	1	3.00 ± 0.00 × 10^5^	9.33 ± 1.15 × 10^3^
Clinafloxacin	5	9.00 ± 1.00 × 10^5^	5.33 ± 3.06 × 10^4^
Clofoctol	35	2.20 ± 0.72 × 10^6^	1.00 ± 0.53 × 10^5^
Miconazole	6	2.07 ± 0.38 × 10^6^	2.13 ± 0.31 × 10^5^
Pentamidine	0.5	2.00 ± 1.00 × 10^6^	2.00 ± 0.00 × 10^5^
Aprepitant	2	1.20 ± 0.17 × 10^7^	9.00 ± 2.65 × 10^5^
Colistin	2	7.33 ± 1.15 × 10^6^	6.67 ± 3.06 × 10^5^
Amifostine	15	3.00 ± 1.00 × 10^6^	5.33 ± 2.23 × 10^5^
Berberine	1	3.40 ± 0.27 × 10^6^	1.00 ± 0.00 × 10^6^

* The beginning CFU for the 5-day old stationary phase *B. henselae* culture was about 2 × 10^8^ CFU/mL.
